# Event-related potential evidence of impaired proactive control in individuals with subthreshold depression

**DOI:** 10.3389/fpsyt.2025.1528316

**Published:** 2025-07-03

**Authors:** Zhijun Wang, Jinsheng Hu, Qi Qiang, Zhihong Liu, Qingshuo Yang

**Affiliations:** School of Psychology, Liaoning Normal University, Dalian, Liaoning, China

**Keywords:** subthreshold depression, cognitive control, proactive control, reactive control, attentional allocation, context updating

## Abstract

**Objective and rationale:**

Cognitive control deficits are considered as central features of cognitive impairments in depression. The dual mechanisms of control (DMC)—proactive and reactive control—can further elucidate the nature of these deficits. However, evidence regarding proactive control in mild depression remains uncertain. This study investigated alterations of DMC and their related neural correlates in subthreshold depression (SD).

**Method:**

Participants with SD were identified through a mental health screening and assigned to the SD group (*n* = 27), while healthy controls (HC) without depressive symptoms were recruited as the control group (*n* = 28). All participants completed the AX-Continuous Performance Task while measuring behavioral (reaction time and accuracy) and electrophysiological responses (cue-P3 and probe-N2/P3). The primary outcome focused on the alteration of proactive control in individuals with SD, assessed through group differences in BX performance and the cue-P3 component. Secondary outcomes encompassed AY trial performance and probe-N2/P3 components, indexing reactive control alteration in individuals with SD.

**Results:**

Slower responses in BX and BY trials were observed for the SD relative to the HC group, indicating the impairment of context processing in individuals with SD. Event-related potential (ERP) results showed that cue-P3 components were less positive for the SD group relative to the HC group, indicating reduced cue utilization and attentional allocation to the cue in individuals with SD. Moreover, the positive correlation between the probe-N2 component and Beck Depression Inventory (BDI) scores implies that individuals with SD may rely more on reactive control.

**Conclusion:**

These findings suggest proactive control deficits in individuals with SD, as evidenced by diminished attentional allocation to the cue and inefficient cue utilization.

## 1 Introduction

Cognitive control operates through two temporally distinct mechanisms: early selection for goal-directed behavior and late correction involving conflict detection and resolution (1, 2). The research on cognitive control in depression has primarily revealed impairments in goal-directed cognitive control in depression (3–5), whereas the other aspect of cognitive control, including conflict detection and resolution, remains poorly understood. To this end, the current study aims to systematically investigate both aspects of cognitive control—specifically goal-directed control and conflict processing—in depressed individuals.

Cognitive control can be distinguished into two distinct modes according to dual mechanisms of control (DMC), namely, proactive and reactive control (6, 7). Proactive control is a form of early selection mechanism that relies on the active maintenance of task-relevant information to minimize interference effects. It would be associated with sustained activation of the lateral prefrontal cortex (PFC) (6, 8). In contrast, reactive control is a late correction mechanism that is employed only as needed, such as after detecting an interference event. It would be reflected in transient activation of the lateral PFC and anterior cingulate cortex (9, 10). This distinction in cognitive control mechanisms can be analogized to everyday preparatory behaviors: proactive control resembles advance planning, such as packing your lunch the night before to avoid midday hunger through preemptive organization, whereas reactive control parallels emergency responses, like grabbing a snack during meetings when sudden hunger strikes, serving as an immediate compensatory measure. This DMC framework provides new perspectives for understanding cognitive dysfunctions in depression. Moreover, numerous studies suggested that proactive and reactive control may have a flexible shift mechanism, with increased proactive control leading to decreased reactive control engagement and *vice versa* (8, 10, 11). In contrast, some studies proposed that two control modes are independent of each other (12, 13).

The AX-Continuous Performance Task (AX-CPT) provides sensitive and specific indices of proactive and reactive control (6, 10). In this task, there are two types of contextual cues (A and B) and probes (X and Y), which combine to form four trial types: AX (70%), AY (10%), BX (10%), and BY (10%). Participants are instructed to give a target response to the probes (i.e., X) in AX trials and to other cases (i.e., AY, BX, and BY) with nontarget response. The majority of trial types are AX trials, inducing a strong bias to give a target response following the A cue or X probe, even on trials other than AX (i.e., AY and BX). An inferior performance in BX trials indicates a reduction in proactive control, due to less use of cue information to prepare a nontarget response (6, 14). Accordingly, an inferior performance in AY trials reflects decreased reactive control. It is due to less efficient processing of nontarget probe (i.e., Y) to overcome a strong bias for a target response after the A cue (8, 15).

Using the AX-CPT, Msetfi et al. (16) suggested that mild depression exerts a detrimental effect on proactive control, as reflected by increased BX errors. In contrast, Masuyama and Mochizuki (17) found that BX error was no different between the mildly depressed and non-depressed participants, and this inconsistent result has been interpreted as the consequence of different cue–probe delays (i.e., 10 vs. 4 s). However, previous AX-CPT studies demonstrated that the length of cue–probe delay might not be critical to modulate context processing (i.e., proactive control) (18–20). Thus, whether proactive control is altered in depressed individuals remains uncertain. Additionally, no studies have examined the effects of depression on the neural correlates underlying proactive and reactive control mechanisms.

This study aimed to investigate the alteration of cognitive control strategies in individuals with subthreshold depression (SD). SD is a developmental prodrome for a major depressive disorder (MDD), in which the number, duration, or quality of symptoms is insufficient to meet the full criteria of MDD (21–23). Given that depressive symptoms impair the PFC-driven top-down cognitive control (3, 4), we hypothesized reduced proactive control in individuals with SD.

## 2 Materials and methods

### 2.1 Participants

The Beck Depression Inventory (BDI-II) (24) was administered to all students during a mental health screen at Liaoning Normal University, and participants with scores of 14 or more were selected to the SD group, since scores above this cutoff indicate the presence of depressive symptomology (24). Healthy controls (HC) were matched for age, gender, and education level. All participants were further assessed with the Diagnostic and Statistical Manual of Mental Disorders via a clinical interview (DSM-5) (25). Participants were excluded on the basis of following criteria: (1) history of manic/hypomanic episode; (2) concurrent or history of personality and bipolar disorders; (3) self-report use of psychotropic substance, e.g., antidepressants; (4) and risk of committing suicide.

A total of 60 participants completed the formal experiment. Five participants were excluded from analysis, because of excessive artifacts in the electroencephalographic recording (<50% trials were valid after artifact rejection). Thus, the data from a total of 55 participants were included in the final analysis (**Table 1**). The sample size was determined using MorePower (Version 6.0). To obtain a large statistical power (0.90) with a 2 group × 4 trial type mixed experimental design, a sample size of 26 per group was necessary based on a moderate effect size (Partial eta squared, *η*_p_^2^) of 0.09 (α = 0.05).

**Table 1:** Demographic characteristics (mean and standard deviations).

Items	SD	HC	Statistics
Sample size	27	28	
Gender (male/female)	11/16	13/15	
Handedness (right/left)	27/0	28/0	
Age	21.37 (2.40)	21.86 (2.38)	*t*(53) = −0.75, *p* = 0.45
BDI-II	22.07 (6.85)	5.93 (4.40)	*t*(53) = 10.44, *p* < 0.001
SDS	0.58 (0.09)	0.44 (0.08)	*t*(53) = 6.08, *p* < 0.001

BDI-II, Beck Depression Inventory-Second Edition; SDS, Self-Rating Depression Scale.

All participants gave their written informed consent prior to involvement in this study and were paid for their participation. The study was approved by the Research Ethics Committee of Liaoning Normal University of China (Approval No. LL2025131), and the research followed the ethical guidelines of the Declaration of Helsinki.

### 2.2 Design and materials

**Figure 1:**
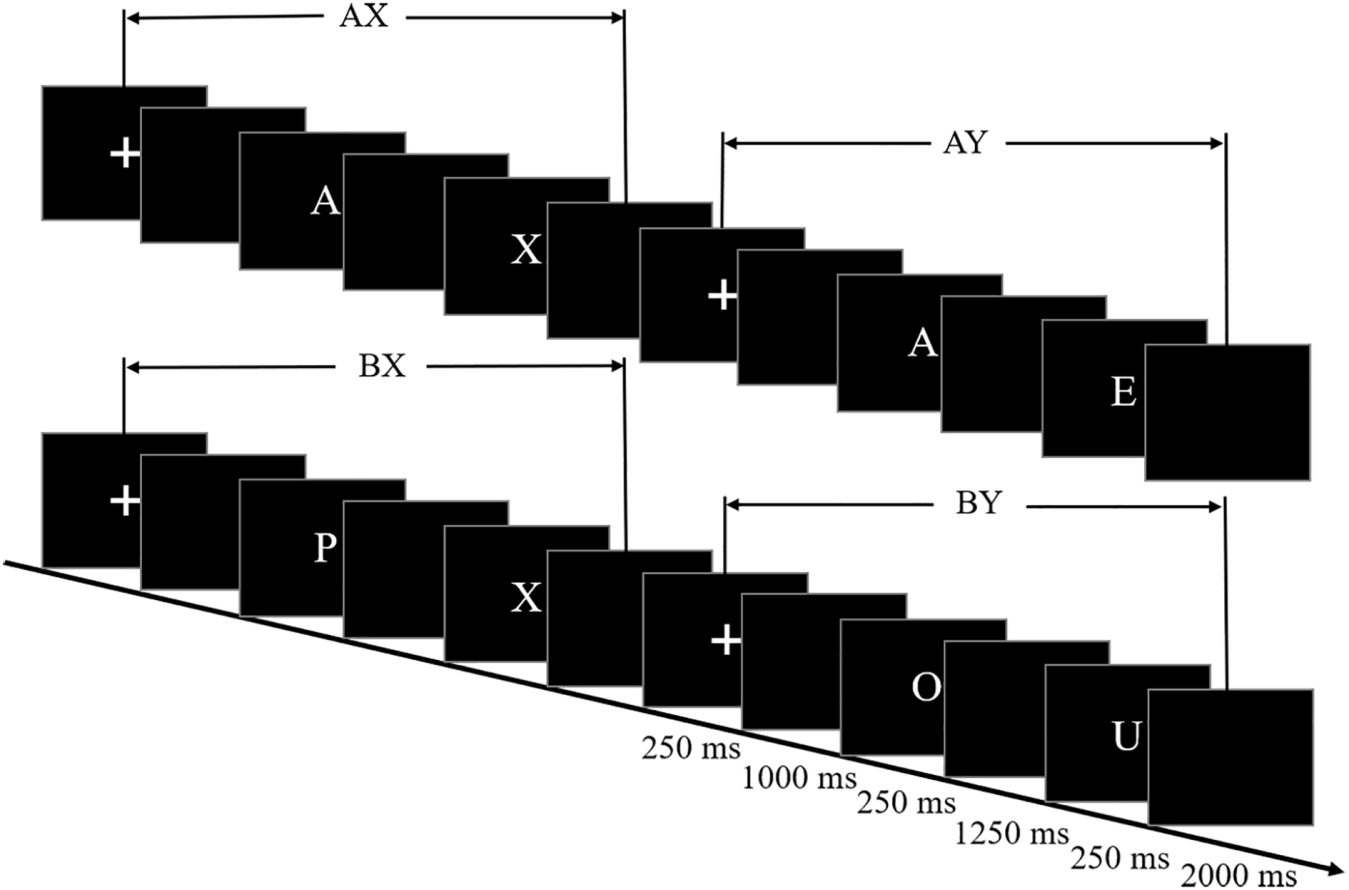
AX-Continuous Performance Test (AX-CPT).

Upon arrival, participants had 20 min to acquaint themselves with the lab environment and filled out the Self-Rating Depression Scale (SDS) (26). All participants completed an AX-CPT (**Figure 1**). In this task, pairs of letters were presented sequentially, with each pair forming a cue–probe sequence. There were four different types of trials: AX, AY, BX, and BY (B cues and Y probes were replaced with the rest of the alphabet, excluding B, H, K, V, W, and Y). Participants gave their response by pressing the button “J” with their right index finger for both the cue and the probe except when an X probe followed an A cue, in which case they had to press the button “F” with their left index finger. Participants were asked to respond as quickly and accurately as possible.

Each trial began with a central fixation for 250 ms, followed by a 1000-ms blank. Then, the cue was presented for 250 ms, followed by a 1,250-ms blank. Subsequently, the probe was presented for 250 ms, followed by a blank screen for 2,000 ms. Consistent with previous event-related potential (ERP) studies adopting the AX-CPT (13, 27–29), a relatively short cue–probe delay (1,500 ms) was employed.

There were six blocks of 500 trials. In each block, AX trials occur in approximately 64%, with the remaining trial types (AY, BX, and BY) accounting for 12% each. These trials are presented on the screen randomly. A brief practice session is conducted prior to the formal experiment (20 trials). Each pair of letters were presented in white 36-point capital bold Helvetica font on a black screen (8, 30, 31). All stimuli were presented on a 19-in Dell monitor and viewed at a distance of 60 cm.

### 2.3 Electrophysiological recording and analysis

A 64-channel electroencephalogram (EEG) recording system was used to record brain electrophysiological activity (Brain Products, GmbH, Germany), with reference on the FCz electrode. An electrode was placed below the right eye to record a vertical electrooculogram (EOG). All interelectrode impedance was kept below 10 kΩ. EEG and EOG were amplified using a 0.05- to 100-Hz bandpass filter and continuously sampled 500 Hz for off-line analysis.

EEG data processing was performed using Brain Vision Analyzer version 2.2 (Brain Product GmbH; Gilching, Germany). For the data analysis, ERPs time locked to the onset of the cue and probe in the AX-CPT task were re-referenced to the average of the left and right mastoids. Eye blinks were removed by using semi-automatic ICA-based ocular correction. Then, the EEGs were filtered at 0.01 Hz (high-pass cutoff) and 35 Hz (low-pass cutoff), slope 24 dB/Oct. The segmentation of cue and probe ranged from −200 ms before to 800 ms after the cue/probe onset. Artifact rejection was applied automatically with an amplitude threshold of ±80 μV. EEGs recorded in the AX-CPT were averaged for each participant, and only trials with correct responses were included in ERP averages. The mean numbers of trials retained after artifact rejection were as follows: SD: A = 349 (22), B = 111 (7), AX = 266 (21), AY = 41 (11), BX = 48 (5), and BY = 49 (5); HC: A = 345 (37), B = 110 (12), AX = 261 (30), AY = 41 (11), BX = 47 (8), and BY = 48 (7).

To further investigate the precise processes by which SD affects cognitive control, ERP measures of proactive and reactive control were investigated in this study. Consistent with previous ERP studies on the AX-CPT (13, 28, 32), the maximum ERP differences across A and B cues for P3 were localized over the parietal scalp. Therefore, the mean amplitudes of three electrodes (P3, P4, and Pz) were chosen for cue-P3 analysis. Since inspection of the data revealed that there were two different peaks in the B cue evoked P3 component, the mean amplitudes of the 240–340 ms and 340–540 ms time windows for the cue were chosen for statistical analysis. The previous study found that the P3 component can be divided into separate subcomponents, including early and late P3 components (33). Moreover, Adamo and Ferber (34) analyzed the early P3 that is derived from the total P3.

Maximum voltage and maximum difference across different trial types for probe-N2 and probe-P3 were shown in frontocentral locations. This is in line with previous ERP studies on the AX-CPT (27, 32, 35). Therefore, three frontocentral scalp electrodes (FC3, FC4, and FCz) were selected for probe-N2 and probe-P3 analysis. The mean amplitudes of probe-N2 (250–400 ms) and probe-P3 (500–700 ms) were calculated from the average of three electrodes for statistical analysis.

### 2.4 Behavioral and electrophysiological data analysis

The mean accuracy of the cue was greater than 98.2% for both A and B cues. A preliminary inspection of the data indicated no accuracy differences across different cues. For the RT analysis, incorrect responses were excluded, as were RTs that were three standard deviations either above or below the mean RTs per cue and trial type for each participant. Finally, 8.8% of the data from the SD group and 11.8% of the data from the HC group were excluded.

Additional behavioral indices reflecting the amount of proactive control were computed: A-cue bias = [Z(hits in AX trials) − Z(false alarms in AY trials)]/2; PBI = (AY−BX)/(AY+BX). PBI was computed in terms of both error rates and RTs on AY and BX trials. A log-linear correction was applied for correct rates and error rates as follows: hit rate = (number of hits + 0.5)/(number of trials + 1); false alarm rate = (number of false alarms + 0.5)/(number of trials + 1) (36). Independent-samples *t*-tests with the factor group (SD vs. HC) were performed on the A-cue bias, PBI-RTs, and PBI-errors. Higher values of these indices indicate more engagement of proactive control.

Repeated-measures analyses of variance (ANOVAs) with group (SD and HC) as between-subject factor and cue (A and B) as within-subject factor were performed on the mean amplitude of 240–340 and 340–540 ms. Repeated-measures ANOVAs with group (SD and HC) as between-subject factor and trial type (AX, AY, BX, and BY) as within-subject factor were performed on RTs and accuracy, and the mean amplitude of probe-N2 and probe-P3, respectively.

## 3 Results

### 3.1 Behavioral results

For the cue RTs (**Table 2**), the main effect of cue was significant, *F*(1, 53) = 150.521, *p* < 0.001, *η*_p_^2^ = 0.740, with slower response for B relative to A cues. Neither the main effect of group nor the group × cue interaction was significant, *F*s < 0.412, *p*s > 0.524.

**Table 2:** Mean RT and accuracy (and standard deviations) in AX-CPT.

Cue/Trial type	RT (ms)		Accuracy (%)	
	SD	HC	SD	HC
Cue A	320.033 (46.764)	331.533 (72.085)	98.326 (2.050)	98.543 (1.769)
Cue B	386.206 (81.681)	391.128 (96.868)	98.248 (2.643)	98.546 (2.174)
AX	368.469 (85.055)	379.167 (67.951)	95.333 (4.069)	95.532 (3.857)
AY	524.717 (97.120)	508.372 (75.950)	76.485 (19.579)	78.393 (15.900)
BX	340.417 (77.753)	294.146 (74.842)	92.656 (8.028)	93.632 (5.665)
BY	334.669 (82.165)	290.029 (61.799)	94.622 (5.608)	95.996 (5.344)

For the probe RTs (**Table 2**), the group × trial type interaction was significant, *F*(3, 159) = 5.129, *p* = 0.008, *η*_p_^2^ = 0.088. Simple effects analysis revealed that (1) compared to the HC group, RTs were slower for the SD group in BX and BY trials, *p*s < 0.029, but not in AX and AY trials, *p*s > 0.489; (2) for both the SD and HC groups, RTs were longer for AY trials than for AX, AY, and BY trials, *p*s < 0.001; RTs were longer for AX trials than for BX and BY trials, *p*s < 0.008, in which AX trials marginally differ from BX trials in the SD group, *p* = 0.054; no difference was found between the BX and BY trials, *p*s > 0.399.

For the accuracy (**Table 2**), the main effect of trial type was significant, *F*(3, 159) = 59.153, *p* < 0.001, *η*_p_^2^ = 0.527. Pairwise comparison revealed that all participants maintain a progressively decreasing accuracy in AY, BX, BY, and AX, *p*s < 0.003, except that the difference between AX and BY was not significant, *p* = 0.811. Neither the main effect of group nor the group × trial type interaction was significant, *F*s < 0.339, *p*s > 0.563.

For proactive control indices (**Table 3**), PBI-RTs were lower in the SD group than in the HC group, *t*(53) = 2.369, *p* = 0.022, *d* = 0.651. No group difference was found on A-cue bias and PBI-errors, *t*s < 0.247, *p*s > 0.806.

**Table 3:** Behavioral indices (standard deviations) for AX-CPT.

Behavior indices	SD	HC
A-cue bias	0.552 (0.061)	0.551 (0.046)
PBI-RTs	0.214 (0.092)	0.273 (0.093)
PBI-errors	0.457 (0.350)	0.478 (0.280)

### 3.2 ERP results

**Figure 2:**
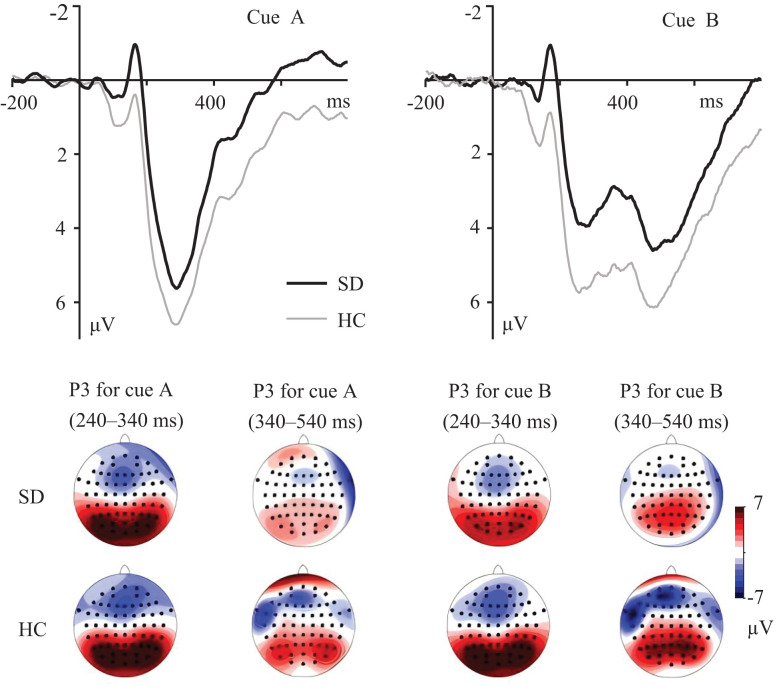
Grand averaged ERPs evoked by A and B cues at parietal electrodes. The topographic maps indicate the distribution of cue-evoked P3 components during 240–340 ms and 340–540 ms.

For the cue-P3 component (**Figure 2**), during the 240–340 ms time windows, the group × cue interaction was significant, *F*(1, 53) = 4.460, *p* = 0.039, *η*_p_^2^ = 0.078. Simple effects analysis revealed that compared to the HC group, the SD group showed less positive ERPs in B cues, *p* = 0.043. No group difference was found in A cues, *p* = 0.248. For both the SD and HC groups, more positive ERPs were found for B compared to A cues, *p*s < 0.003.

During the 340–540 ms time windows (**Figure 2**), the main effect of group was significant, *F*(1, 53) = 4.559, *p* = 0.037, *η*_p_^2^ = 0.079, with more positive ERPs for the HC group compared to the SD group. The main effect of cue was significant, *F*(1, 53) = 60.619, *p* < 0.001, *η*_p_^2^ = 0.534, with more positive ERPs for the B compared to the A cues. The group × cue interaction was not significant, *F*(1, 53) = 0.239, *p* = 0.627.

**Figure 3:**
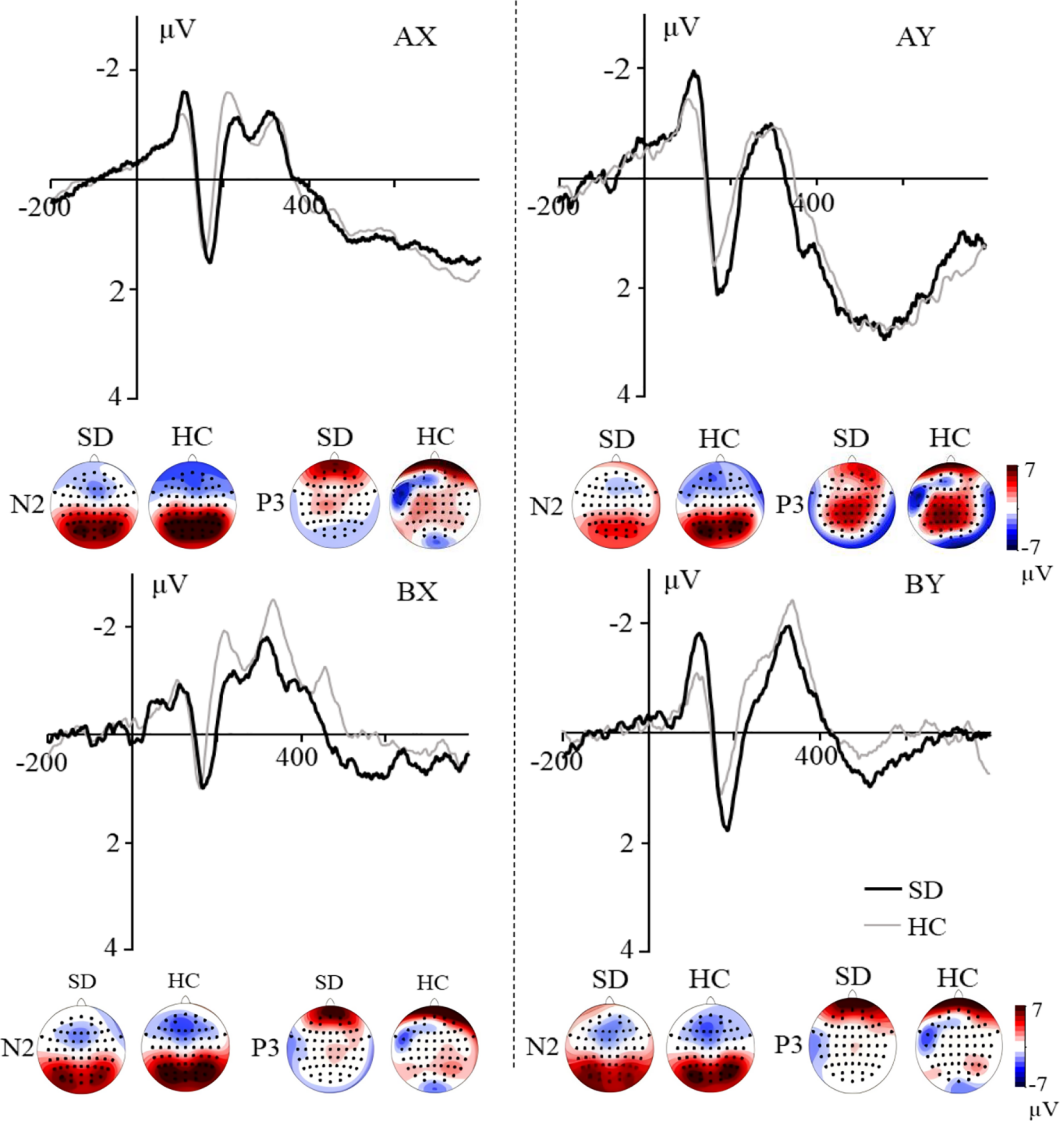
Grand averaged ERPs evoked by AX, AY, BX and BY at frontal electrodes. The topographic maps indicate the distribution of N2 and P3 components evoked by the probe.

For the probe-N2 component (**Figure 3**), the main effect of trial type was significant, *F*(3, 159) = 9.810, *p* < 0.001, *η*_p_^2^ = 0.156. Pairwise comparison revealed that ERPs were more negative for BX and BY trials compared to the AX and AY trials, *p*s < 0.004. There was no significant difference between the AX and AY trials, *p* < 0.089, and no significant difference was found between BX and BY trials, *p* = 0.631. Neither the main effect of group nor the group × trial type interaction was significant, *F*s < 0.422, *p*s > 0.699.

For the probe-P3 component (**Figure 3**), the main effect of trial type was significant, *F*(3, 159) = 24.925, *p* < 0.001, *η*_p_^2^ = 0.320. Pairwise comparison revealed that more positive ERPs were evoked for AY compared to AX, BX, and BY trials, *p*s < 0.001; more positive ERPs were evoked for AX compared to BX and BY trials, *p*s < 0.007. No difference was found between BX and BY trials, *p* = 0.437. Neither the main effect of group nor the group × trial type interaction was significant, *F*s < 0.498, *p*s > 0.697.

### 3.3 Correlational analyses

Pearson correlation between depressive severity (BDI score) and ERP indices was determined (only for participants in the SD group). BDI score was positively correlated with the probe-N2 component in AY trials, *r* = 0.382, *p* = 0.049. No significant correlations were found between BDI score and probe-N2 in AX, BX, and BY trials, *r*s < 0.345, *p*s > 0.078. BDI score was not significantly correlated with cue-P3 and probe-P3, |*r*|s < 0.181, *p*s > 0.367.

## 4 Discussion

The present study investigated the influences of SD on proactive and reactive control. Compared with the HCs, participants with SD reported higher levels of depressive symptoms (BDI-II and SDS scores). The SD group comprises individuals experiencing significant depressive symptoms that fall below the diagnostic threshold for MDD. This subclinical presentation constitutes a documented risk factor for subsequent MDD development (21–23). By focusing on this population, we can examine neural correlates without confounding effects from clinical interventions (37). We hypothesized that if proactive control decreased in individuals with SD, an inferior performance in BX trials is expected for the SD group compared to the HC group. Decreased cue evoked ERPs were expected for the SD compared to the HC group. Results indicate a reduced proactive control in individuals with SD, as reflected by reduced cue utilization and attentional allocation to the contextual cue.

Compared to the HC group, slower RT in BX and BY trials was found for the SD group. Intact context maintenance would lead to faster responses in the BX and BY trial, because a nontarget response has been prepared before the probe onset (6, 20). The slower response in BX trials could be attributed to inefficient processing of cue information (reduced proactive control) or increased focus on probe (enhanced reactive control) in individuals with SD (6, 18, 27). However, increased reactive control in participants with SD is not supported by the results in AY trials. Thus, the slower response to BX and BY trials might suggest a reduction in context processing, indicating impaired proactive control in individuals with SD. This is consistent with the finding of Msetfi et al. (16) in which reduced context processing was observed in depressed participants. Furthermore, as context maintenance might be independent of context activation/updating during context processing, varying lengths of cue–probe delay might not be critical in detecting context processing (18–20). Accordingly, impaired proactive control in individuals with SD could be attributed to blunted context activation/updating.

ERP measures have provided further insight into the underlying neural correlates of proactive and reactive control in individuals with SD. During the 240–340 ms time windows, reduced cue-P3 amplitudes in B cues were found for the SD group. Parietal P300 amplitude indexes the amount of attentional resources engaged during task performance (38–40). During the AX-CPT, P3 amplitude evoked by the B cue has been associated with allocating attentional resources to the salient stimuli, since the B cue is presented with lower frequency (28, 32, 35). A reduced cue-P3 component suggests that less amount of attentional resources were allocated to the contextual cue, indicating reduced proactive control in individuals with SD.

During the 340–540 ms time windows, reduced cue-P3 components were observed for the SD compared to the HC group. This cue-P3 component is thought to reflect context updating of task-relevant information in working memory (27, 28, 41), and thereby indicates cue utilization during the AX-CPT (13, 42). The present findings suggest impaired proactive control in individuals with SD resulting from inefficient utilization of contextual cue. This result may gain further support from the slow response in BX and BY trials in SD participants. Moreover, peak amplitude of cue-P3 was not evoked in the A cue during the 340–540 ms time windows. This might be attributed to the highest frequency of the AX trail, in which case updating the contextual cue was not necessary for the frequent appearance of A cues (8, 43). Overall, the analysis of the cue-P3 component is in line with previous studies in which the P3 component could be further separated into early and late subcomponents (33, 34, 44). Numerous studies suggested that the parietal P3 component indexes attentional resource allocation and subsequent memory processing (40, 45, 46). Thus, reduced cue-P3 during the 240–340 ms and 340–540 ms time windows in individuals with SD may indicate a decreased focus on cue, and thereby inefficient context processing during the AX-CPT.

Taken together, this study found that individuals with SD exhibit reduced proactive control due to impaired attentional allocation and subsequent working memory processing. Braver et al. (10) found that the activation dynamics in the lateral PFC could shift from cue-based to probe-based activation after penalty incentives in younger adults, indicating reduced proactive control leading to compensatory enhancement of reactive control. In this study, decreased proactive control did not elicit enhanced reactive control, with no group difference observed in behavior/ERP indices. However, BDI scores were positively correlated with the probe-N2 component in AY trials. The probe-N2 component has been thought to be associated with conflict detection, and increased probe-N2 elicited by AY trials reflects enhanced reactive control (27, 32, 35). The DMC framework hypothesized that proactive and reactive control could flexibly shift from one to another, with reduced proactive control leading to enhanced reactive control and vice versa (8, 10, 11). The observed positive correlation between BDI score and probe-N2 amplitude may suggest that individuals with SD exhibit increased reliance on reactive control when experiencing deficits in proactive control. Moreover, previous research suggested that proactive control implementation demands greater cognitive effort compared to reactive control (6). Individuals with MDD have been found to have cognitive effort deficits (47), which may lead them to rely more on reactive control strategy that require less cognitive effort.

Previous research has primarily documented impairments in goal-directed cognitive control among individuals with MDD (3–5), reflecting reduced proactive control within the DMC framework. This diminished proactive control is largely attributable to deficits in attentional and working memory processes (48, 49). Although SD presents with less severe symptoms than MDD, studies consistently demonstrate SD-related reductions in attention and working memory (50, 51). Current lines of evidence indicate that proactive control is impaired across the depression spectrum, from SD to clinical (MDD) states. Furthermore, our findings indicate that worsening depressive symptoms may coincide with compensatory increases in reactive control, an effect potentially more pronounced in MDD.

This is the first study that explored the neural correlates of DMC in individuals with SD. Previous research investigating DMC alterations in mild depression found that depressed participants exhibit lowered or comparable proactive control relative to the control group, as reflected by behavioral performance in BX trials (16, 17). By recording electrophysiological data, this study provides novel evidence that reduced proactive control in depressed individuals may be due to impaired attentional allocation and working memory processes. Notably, obtained results revealed that elevated BDI score was accompanied by increased automatic conflict detection, which may indicate heightened reactive control in individuals with SD. Taken together, these findings indicate that flexibility in DMC may be manifested as increased automatic conflict detection (reactive control) in individuals with SD, resulting from deficits in proactive control.

Trade-offs between proactive and reactive control are ubiquitous in our everyday lives. Proactive control facilitates the sustained maintenance of task goals between intention formation and behavioral implementation (6). For example, a student plans to finish a paper draft within 1 day. Using proactive control strategy, they break the task into hourly subtasks (writing 100 words each hour) and decline social events that might disrupt progress, ensuring task goal achievement. In contrast, an SD student with impaired proactive control may create the same task goal but fail to maintain it. The goal becomes episodic storage and only retrieved by a salient trigger event, which may result in missing the deadline. Academic/work challenges for individuals with SD with impaired proactive control are often linked to depleted cognitive resources and reduced motivation (4), resulting in their inability to schedule activities.

This study provides valuable implications for intervention strategies of cognitive impairments in depression. In line with previous studies showing that cognitive control requires goal-directed attention and working memory processes (6, 46), our findings further indicate reduced proactive control in individuals with SD potentially attributed to impaired attentional allocation and working memory updating. Thus, targeting attention and working memory through cognitive training (e.g., mindfulness and integrative body–mind training) may serve as an effective intervention to restore proactive control in depressed individuals. Moreover, converging evidence from incentive manipulations indicates increased proactive control under reward conditions (6, 10). Accordingly, utilizing reward anticipation mechanisms may improve proactive control in depressed individuals.

There are two limitations in this study. Firstly, there is a lack of observed group difference in reactive control indices. This may be attributable to subthreshold depressive severity being insufficient to modulate reactive control. Moreover, proactive and reactive control are associated with different activation dynamics of the lateral PFC (10). Spatial information on lateral PFC activation may help elucidate proactive and reactive control engagement in depression. Future research could further investigate reactive control modulation in MDD and employ additional techniques [e.g., functional magnetic resonance imaging (fMRI) and functional near-infrared spectroscopy (fNIRS)] to examine the underlying neural activation. Secondly, this study did not assess anxiety levels in the SD group. Trait and state anxiety has been found to be associated with reduced sustained but increased transient activation in the lateral PFC during the n-back task (52, 53), which may modulate proactive and reactive control. In reality, individuals with anxiety symptoms may experience greater difficulty maintaining goal-relevant information in working memory, as this capacity becomes consumed by intrusive unrelated thoughts (e.g., worries, rumination, or unpredictable threats) (5). Hence, further research should incorporate the State-Trait Anxiety Inventory (STAI) in participant screening to exclude individuals with elevated anxiety (e.g., students with scores ranked above 75% of the distribution) (37), thereby reducing potential confounding effects of anxiety comorbidity.

In conclusion, the present study provides some evidence of proactive control deficits in individuals with SD. Specifically, slower RTs in BX and BY trials were observed for the SD than for the HC group, suggesting inefficient context processing. Furthermore, reduced cue-P3 components were found for the SD relative to the HC group, indicating reduced allocation of attentional resource to the cue and inefficient utilization of cue information in individuals with SD.

## Data Availability

The raw data supporting the conclusions of this article will be made available by the authors, without undue reservation.
